# Relationships between walking speed, activities and participation in people with chronic stroke in Burundi

**DOI:** 10.4102/sajp.v78i1.1800

**Published:** 2022-10-31

**Authors:** Félix Nindorera, Ildephonse Nduwimana, Alexis Sinzakaraye, Yannick Bleyenheuft, Jean-Louis Thonnard, Oyéné Kossi

**Affiliations:** 1 Institute of Neuroscience, Catholic University of Louvain, Brussels, Belgium; 2 National Center of Reference in Physical Therapy and Medical Rehabilitation, University Hospital Roi-Khaled, Bujumbura, Burundi; 3 National School of Technicians in Public Health and Epidemiological Surveillance, Université de Parakou, Parakou, Benin

**Keywords:** activity limitations, low-income settings, social participation, stroke, walking speed

## Abstract

**Background:**

Reduced walking speed because of a stroke may limit activities of daily living (ADLs) and restrict social participation.

**Objectives:**

To describe the level of balance impairment, activity limitations, and participation restrictions and to investigate their relationship with walking speed in Burundians with chronic stroke.

**Methods:**

This cross-sectional study involved adult stroke survivors. Walking speed, balance, ADLs and social participation were assessed with the 10-meter walk test (10 mWT), the Berg balance scale (BBS), the activity limitation stroke scale and the participation measurement scale, respectively. In order to determine ambulatory independence status, participants were stratified into three walking speed groups (household ambulation, limited ambulation and full-community ambulation), based on the Perry classification.

**Results:**

Fifty-eight adults (mean age 52.1 ± 11.4 years) with chronic stroke were included in our study. Most participants had severe balance impairments (median BBS score, 27). Their mean (± standard deviation [SD]) walking speeds, ADL levels and social participation levels were 0.68 ± 0.34 m/s, 50.8% ± 9.3% and 52.8% ± 8.6%, respectively. Walking speed correlated moderately with balance (rho = 0.5, *p* < 0.001) and strongly with ADL level (*r* = 0.7, *p* < 0.001) but not with participation level (*r* = 0.2, *p* = 0.25).

**Conclusion:**

Using socio-culturally suitable tools, our study showed that walking speed correlates robustly with balance and ADL ability, but not with social participation, in Burundi, a low-income country.

**Clinical implications:**

Exercises targeting walking speed would be very useful for people with chronic stroke living in low-resource countries, in order to promote their functional independence.

## Introduction

Stroke is the second leading cause of death and a major cause of disability worldwide (Katan & Luft 2018). Disability because of stroke is still very common up to three years after the event (Kossi et al. 2021). In a 2016 meta-analysis, the total prevalence rate for stroke in Africa was estimated to be 3.5 cases per 1000 people, with an annual increasing trend of 12.0% (Ezejimofor et al. 2016). Relative to high-income countries, stroke affects more young people in middle- and low-income countries, and the affliction of younger people with stroke places an economic burden on such countries (Katan & Luft 2018). Furthermore, the highest incidence, prevalence and case fatality rates of stroke in the world have been reported in Africa (Adoukonou et al. 2021).

The World Health Organization’s international classification of functioning, disability and health (ICF) recognises functional disability as multidimensional and involving nonlinear concepts of the interaction between a person’s health condition and contextual factors. Commonly, stroke survivors experience activity limitations and social participation difficulties, even following a relatively good recovery from impairments (Van der Zee et al. 2013). The reported prevalence of social participation restrictions among stroke patients ranges from 12% to 64% (Adoukonou et al. 2018; Kossi et al. 2019). The improvement of ICF components depends on several factors, including the national economic situation, access to rehabilitation and cultural beliefs (Lekander et al. 2017; Mbada et al. 2019; Mendis 2013). In low-income countries, like Burundi, understanding the challenges faced by people who have suffered a stroke would be useful in identifying the long-term issues that care-based services for stroke will need to address (Ch’ng, French & McLean 2008).

Walking is often severely affected after stroke and the high prevalence of balance impairments remains a problem (Kossi et al. 2021; Pinto et al. 2014). People tend to reduce their walking speed to prevent the risk of falling, and a slow walking speed may limit one’s ability to perform the activities of daily living (ADLs) and limit one’s participation in family and community life (Faria-Fortini et al. 2019; Wang et al. 2020). In a sample of 147 patients, Perry and colleagues (1995) defined functional walking status according to three community walking speed categories: household ambulation (speed < 0.4 m/s, indicating severe walking limitations), limited-community ambulation (speed in the range of 0.4 m/s – 0.8 m/s, indicating moderate walking limitations) and full-community ambulation (speed > 0.8 m/s, reflecting slight or mild walking limitations). Although this classification comes from a high-income context, it can provide information on the functional status of walking in low-income countries. Factors shown to influence ADL abilities and social participation include age, gender, use of medication, time after stroke, number of physiotherapy sessions attended, walking speed, balance and motor function (Faria-Fortini et al. 2018; Kossi et al. 2019). However, consistently, walking speed has been shown to be the best indicator of walking performance (Faria-Fortini et al. 2019). Thus, physical outcomes, such as walking speed, should not be assessed in isolation when considering community ambulation post-stroke (Durcan, Flavin & Horgan 2016). To date, the relationship between walking speed and stroke outcomes has been studied in developing and high-income countries; but to the best of our knowledge, no studies have been conducted in Burundi (Faria-Fortini et al. 2019; Wang et al. 2020). Therefore, our study aimed (1) to describe balance impairments, ADL levels and social participation levels of chronic stroke patients in Burundi and (2) to investigate their relationship with walking speed.

## Methods

A cross-sectional study was conducted at the National Center of Reference in Physical Therapy and Medical Rehabilitation, University Hospital Roi-Khaled, Bujumbura between September 2019 and February 2020. Our study population consisted of people suffering from a stroke in Burundi at the time of this study. Potential participants were identified by consulting admission records in hospitals and rehabilitation centres. Data for all potential participants were screened to ensure that they met our inclusion criteria: a clinical diagnosis (imaging or neurologist’s diagnosis) of a primary or recurring unilateral stroke, chronic stroke stage (time since stroke ≥ 6 months), age ≥ 18 years and ability to walk 10 m with or without an assistive device. Participants with a major cognitive impairment (community screening interview for dementia score ≥ 7) (Hall et al. 2000) and those who had other neurological impairments with permanent degenerative damage (such as Parkinson’s disease or Alzheimer’s disease) were excluded.

### Sampling strategy

A purposive sampling technique was used to recruit participants. The minimum sample size was determined by considering a 5% margin of error, a 95% confidence level and 0.4% stroke prevalence (Kim et al. 2015). The total sample size was proportional to the five public and private hospitals in Bujumbura city, based on their total number of beds. The minimum sample size required at the University Hospital Roi-Khaled of Bujumbura was then estimated at 53 participants.

### Procedure and outcome measurements

Questionnaires were administered in face-to-face interviews and clinical tests were conducted in rehabilitation settings. A trained physiotherapist with 4 years of clinical experience collected the data.

#### Walking speed

Walking speed was measured with the 10-meter walk test (10 mWT). The 10 mWT measures comfortable or maximal walking speed. Participants were asked to walk comfortably on a 14-m marked pathway, and they were timed over the middle 10-m distance to record their comfortable walking speeds (Bohannon 1997). The average speed over three trials was recorded. Based on their walking speeds, the participants were stratified into three walking status groups using Perry’s classification (Perry et al. 1995), as follows: household ambulation (< 0.4 m/s), limited-community ambulation (0.4 m/s – 0.8 m/s) and full-community ambulation (> 0.8 m/s).

#### Balance

The Berg balance scale (BBS) was used to assess balance impairment and fall risk. The BBS was developed to measure balance in elderly individuals (Berg et al. 1992). It consists of 14 items that are each scored on a scale of 0 (unable to do the task) to 4 (able to complete the task fully). The maximum total score on the test is 56 (a higher score means better balance). The items include simple mobility tasks (e.g., transfers, standing unsupported, sit-to-stand) and more difficult tasks (e.g., tandem standing, turning 360°, single-leg stance). The BBS is valid, reliable and responsive, and a BBS score ≤ 42 has been previously identified as a predictor of risk of falling (Tilson et al. 2012).

#### Activity limitations

Activities of daily living limitations were evaluated with the ACTIVLIM-stroke scale, a 20-item Rasch-built stroke-specific scale designed to measure functional independence (Batcho, Tennant & Thonnard 2012). This scale has been validated cross-culturally in both African and European contexts. Participants were asked to rate each task on a three-point scale: impossible (0), difficult (1) and easy (2). Total ACTIVLIM-stroke raw scores (ranging from 0 ‘more limited’ to 40 ‘normal’) were subjected to logit and centile metric transformation, allowing parametric statistics. Higher scores indicate less difficulty with ADLs.

#### Participation restrictions

Social participation restrictions were assessed with the participation measurement scale (PM-scale), a 22-item Rasch-built stroke-specific scale for measuring social participation (Kossi et al. 2018; Kossi & Thonnard 2018). The PM-scale is a self-reported questionnaire scale that addresses one’s overall involvement in daily situations and presents good psychometric qualities in stroke survivors in Africa. Participants were asked to rank their perceived participation on a three-point scale: not at all (0), weakly (1) or strongly (2). Total raw scores on the PM-Scale range from 0 (more restricted) to 44 (normal), while the linear measurement logits range from -6.56 to 6.51, corresponding to 0–100 centiles. Higher values indicate more participation.

We converted raw scores from these two questionnaires (ACTIVLIM-Stroke and PM-Scale) into centiles, using an online tool at www.rehab-scales.org.

### Statistical analysis

Descriptive statistics were used to summarise our study sample’s characteristics. Continuous quantitative variables with a normal distribution are reported as means with standard deviations (SDs), while ordinal or non-normal distributed data are reported as medians with interquartile ranges (IQRs). Scores were compared across groups with analysis of variance (ANOVA) and Bonferroni post hoc tests, as indicated. Spearman’s correlation (rho) analysis was used for dichotomous and ordinal variables, whereas Pearson’s correlation (*r*) analysis was used for continuous variables. The magnitude of each relationship was interpreted based on the Munro correlation descriptors: very low (0–0.25), low (0.26–0.49), moderate (0.50–0.69), high (0.70–0.89) and very high (0.90–1.00) (Martin 2004). Statistical Package for the Social Sciences (SPSS) for Windows (version 27, IBM) was used for all statistical analysis. The significance threshold was set at *p* < 0.05.

### Ethical considerations

Potential participants were informed about the objectives of our study and were then invited to provide consent. Our study was conducted in accordance with the Declaration of Helsinki and approved by the Burundi National Ethics Committee under the number: CNE/25/2019.

## Results

### Participant characteristics

**FIGURE 1:**
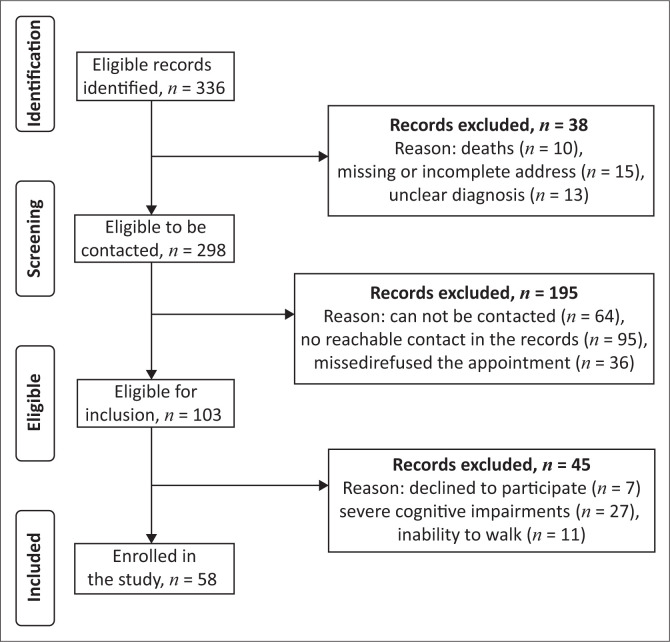
Flow chart of participant eligibility and inclusion.

The sociodemographic and clinical characteristics of our study participants, grouped according to walking speed status, are presented in Table 1. The participant enrolment process is shown in Figure 1. Briefly, of a total of 336 individuals with stroke who were identified in the hospital admission registries and rehabilitation centres, 58 with confirmed chronic stroke (46 men, mean age = 52.1 ± 11.4) met our inclusion criteria and were included in our study. Post-stroke intervals ranged from 6 to 120 months (median, 18.5 months). With respect to walking speed status, 15 participants (26%) were classified as household ambulators (walking speed, 0.26 m/s ± 0.09 m/s); 25 (43%) were limited-community ambulators (walking speed, 0.64 m ± 0.11 m) and 18 (31%) were full-community ambulators (walking speed, 1.11 m/s ± 0.13 m/s).

**TABLE 1:** Socio-demographic and clinical characteristics of the sample, stratified by walking speed status. Sub-groups according to the walking speed on 10 mWT (m/s).

Variables	Total (*N* = 58) %					< 0.4 (*n* = 15)					0.4–0.8 (*n* =25)					> 0.8 (*n* = 18)					*p*
	*n*	%	Mean ± SD	Median	IQR	*n*	%	Mean ± SD	Median	IQR	*n*	%	Mean ± SD	Median	IQR	*n*	%	Mean ± SD	Median	IQR	
**Age, years**			52.1 ± 11.4	-	-	-	-	53.2 ± 9.4	-	-	-	-	54.5 ± 10.2	-	-	-	-	47.9 ± 13.7	-	-	0.16†
**Distribution according to the median age (years)**																					
≤ 50	27	46.6	-	-	-	6	22	-	-	-	10	37	-	-	-	11	41	-	-	-	0.33‡
> 50	31	53.4	-	-	-	9	29	-	-	-	15	48.4	-	-	-	7	22.6	-	-	-	
**BMI**	-	-	25.5 ± 4.6	-	-	-	-	26.5 ± 6.4					25.6 ± 3.9	-	-			24.4 ± 3.6	-	-	0.41†
**Gender**																					
Male	46	79.3	-	-	-	11	23.9	-	-	-	21	45.7	-	-	-	14	30.4	-	-	-	0.71‡
Female	12	20.7	-	-	-	4	33.3	-	-	-	4	33.3	-	-	-	4	33.4	-	-	-	
**Post-stroke duration, month**	-	-	-	18.5	9–33	-	-	-	11	9–24	-	-	-	25	8.5–36.5	-	-	-	18	10–29	0.70‍§
**Distribution according to time since stroke (months)**																					
6–12	24	41.4	-	-	-	8	33.3	-	-	-	10	41.7	-	-	-	6	25	-	-	-	0.08‡
13–24	15	25.9	-	-	-	4	26.7	-	-	-	3	20	-	-	-	8	53.3	-	-	-	
> 24	19	32.8	-	-	-	3	15.8	-	-	-	12	63.2	-	-	-	4	21.1	-	-	-	
**Affected side**								-					-					-			
Right/ Left	30/28	-	-	-	-	5/10	-	-	-	-	14/11	-	-	-	-	11/7		-	-	-	0.24‡
**MRS**	-	-	-	3	2–3	-	-	-	-	-	-	-	-	-	-	-	-	-	-	-	
**Stroke type**								-					-					-			
Ischaemic	25	43.1	-	-	-	8	32	-	-	-	9	36	-	-	-	8	32	-	-	-	0.83‡
Haemorrhagic	26	44.8	-	-	-	6	23	-	-	-	12	46	-	-	-	8	31	-	-	-	
Undetermined	7	12.1	-	-	-	1	14.3	-	-	-	4	57.1	-	-	-	2	28.6	-	-	-	
**Walking speed (m.s^−1^)**	-	-	0.68 ± 0.34	-	-	-	-	0.26 ± 0.09	-	-	-	-	0.64 ± 0.11	-	-	-	-	1.11 ± 0.13	-	-	< 0.001†

10 mWT, 10 metre walk; SD, standard deviation; IQR, interquartile ranges; MRS, modified Rankin scale; BMI, body mass index;

† , one way-ANOVA;

‡ , chi-square tests;

§ , Kruskal–Wallis tests.

### Impairments, activity limitations and participation restrictions

Table 2 provides the participants’ overall outcomes, as well as the distribution according to their walking speed level.

**TABLE 2:** Descriptive data of walking speed, balance, activity limitations and participation. Sub-groups according to the walking speed on 10 mWT (m/s).

Variables	Total (*n* = 58)			Household ambulation			Limited ambulation			Full-community ambulation		
				< 0.4 (*n* = 15)			0.4–0.8 (*n* = 25)			> 0.8 (*n* = 18)		
	Mean ± SD	Median	IQR	Mean ± SD	Median	IQR	Mean ± SD	Median	IQR	Mean ± SD	Median	IQR
BBS scores	-	27	-	-	16	-	-	28	-	-	32.5	-
	-	-	18–33	-	-	10–24	-	-	21.5–32	-	-	26–36
Activilim-Stroke	51% ± 9%	-	-	42.3 ± 8.6	-	-	50.1 ± 7.2	-	-	58.4 ± 5.2	-	-
PM-Scale	52% ± 8%	-	-	47.5 ± 9.2	-	-	54.6 ± 9.2	-	-	54.8 ± 5.5	-	-

SD, standard deviation; BBS, Berg Balance Scale; PM-scale, Participation Measurement scale; IQR, interquartile ranges; 10 mWT, 10 meter walk test.

#### Balance

**FIGURE 2:**
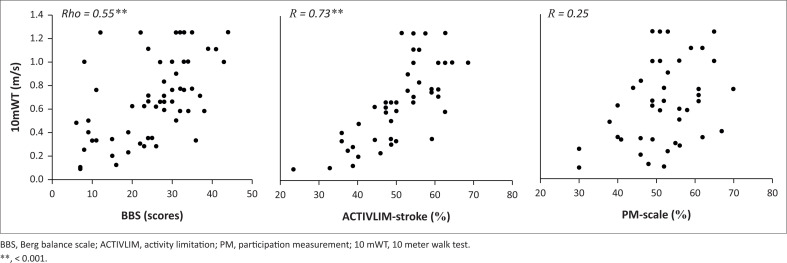
Association between walking speed and balance (Berg balance scale scores), activity limitation (stroke scale) and social participation (participation measurement-scale).

The median score (IQR) on the BBS for the whole sample was 27 (18–33). Fifty-five participants (95%) scored below 40 on the BBS. A moderate, but significant, correlation was found between walking speed and balance (rho = 0.5, *p* < 0.05; Figure 2). A Kruskal–Wallis test showed significant differences between the three walking speed groups on the BBS (*p* = 0.001).

#### Activity limitations

**FIGURE 3:**
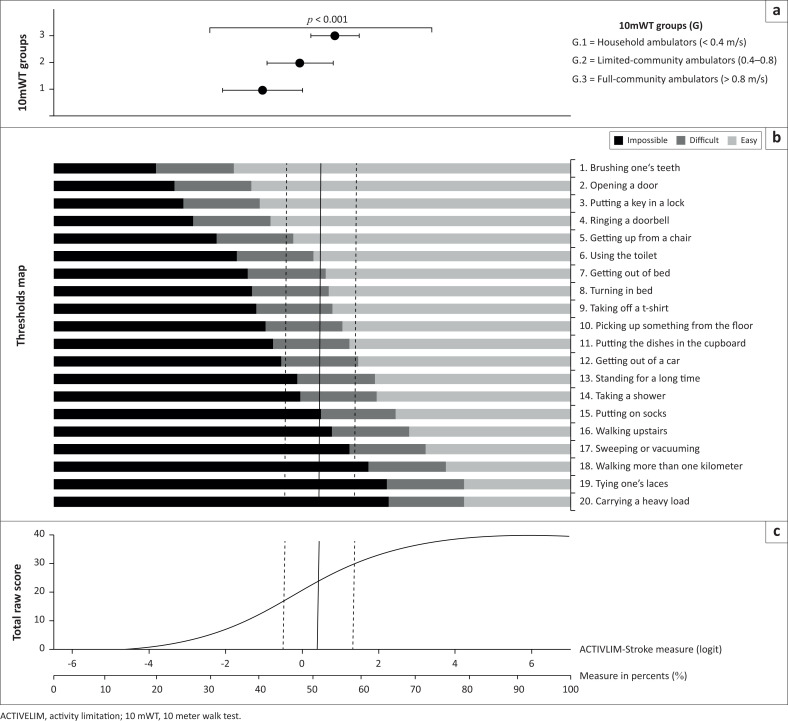
Graph showing activity limitation outcome.

The ADL limitations, according to ACTIVLIM-Stroke scale scores, which were associated with each walking speed status group, are presented in Figure 3, in a graph illustrating the threshold score ranges for the groups for each item, as well as the relationship between ordinal raw scores and the corresponding linear measurements (in logits and in %, according to Batcho et al.’s calibration (Batcho et al. 2012). The overall mean ACTIVLIM-Stroke percentage for the whole sample was 50.8% ± 9.3%, indicating that the participants were able to perform the first eight items easily (from brushing one’s teeth to turning in bed) but could not perform the five most difficult items (walking upstairs, tying one’s laces, carrying a heavy load; sweeping or vacuuming, walking more than one kilometre).

Upon closer inspection, we found ADL differences between the walking speed status groups:

1. Household ambulators (speed < 0.4 m/s) had a mean percentage score of 42% ± 8%, indicating that they performed the two easiest items easily (brushing one’s teeth and opening a door). They were able to perform, with difficulty, several activities requiring some strength and motor co-ordination, including ‘getting up from a chair’, ‘taking off a t-shirt’ and ‘getting out of a car’. Overall, they were unable to perform most of the listed ADLs.
2. Limited-community ambulators (04 m/s – 0.8 m/s) had a mean percentage score of 50% ± 7%, meaning that they performed the five easiest items but had difficulty with activities such as getting out of a car, dressing and standing for a long time.
3. Full-community ambulators (> 0.8 m/s) had a mean percentage score of 58% ± 5%, indicating that most patients could perform basic tasks (‘brushing one’s teeth’, ‘taking off a t-shirt’ and ‘using the toilet’) easily while being able to perform activities involving walking (‘walking more than one kilometre’ and ‘walking upstairs’) with difficulty. They were unable to ‘carry a heavy load’ or to perform some activities requiring manual dexterity, such as ‘tying one’s laces’.

A one-way ANOVA test showed that walking speed had a significant relationship with ADL limitations (ACTIVILIM-Stroke scores, *p* < 0.001), with significant differences in all pair-wise inter-group comparisons (Bonferroni post hoc; *p* < 0.001).

#### Participation restrictions

**FIGURE 4:**
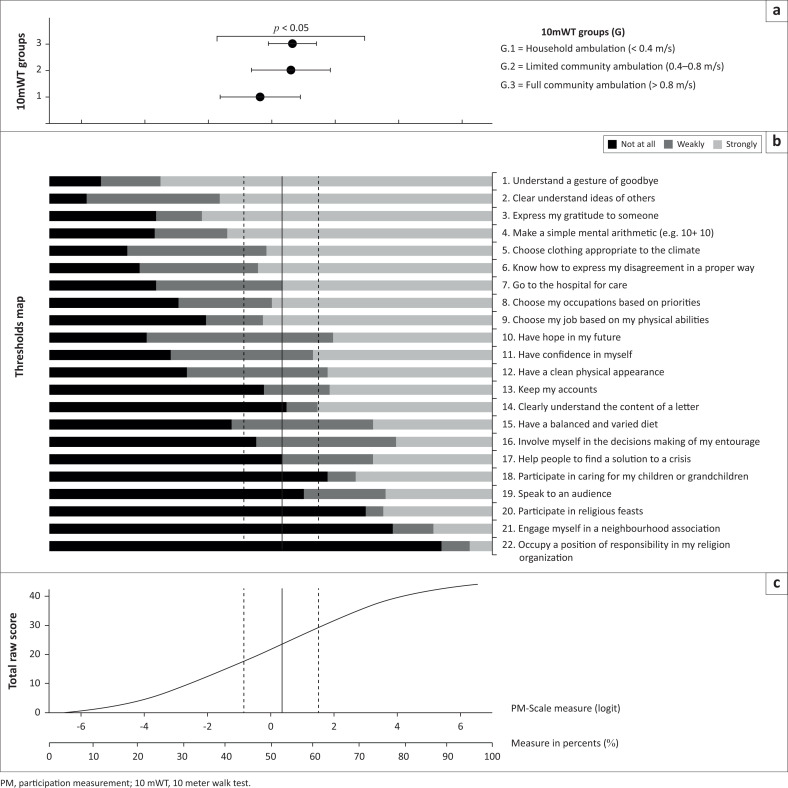
Graph showing social participation outcome.

Overall, walking speed as a continuous variable did not correlate significantly with social participation (*R* = 0.2, *p* > 0.05; Figure 2). Figure 4 illustrates the mean ± SD of the social participation in each walking speed level together with a threshold graph, mapping the expected score ranges for the groups for each item, with an illustration of the relationship between the ordinal raw scores and the corresponding linear measurements (in logits and in %), using Kossi et al.’s calibration (Kossi et al. 2018). The sample’s mean participation percentage score was 52% ± 8%, indicating that patients were strongly involved in the 10 least demanding items (from understanding a goodbye gesture to having hope for the future), while social activities involving leadership and inter-personal interactions were difficult (participating in religious feasts, speaking to an audience and occupying a position of responsibility in a regional organisation).

Upon closer inspection, we found differences in social participation restrictions across the walking speed groups:

1. Household ambulators had a mean percentage score of 47% ± 9%, indicating that they only had regular involvement in simple social activities related to general tasks and demands, communication, and learning and applying knowledge (such as ‘understanding a gesture of goodbye’ and ‘doing simple mental arithmetic’). They had limited involvement in intermediate activities such as ‘choosing clothing’ and showed weak results for ‘having confidence’. They were not involved in many of the major activities in life involving interpersonal interactions and relationships, nor in community, social and civic activities.
2. Limited-community ambulators (mean, 54% ± 9%) and full-community ambulators (mean, 54% ± 5%) had similar levels of social participation, including strong involvement in the 10 least demanding items, weak involvement in the following seven items and an inability to participate in tasks such as ‘participating in caring for children’, ‘participating in religious feasts’, ‘speaking to an audience’ and ‘occupying a position of responsibility’.

A one-way ANOVA test indicated a strong connection between walking speed and social participation (PM-Scale scores, *p* ≤ 0.05). Bonferroni post hoc tests showed that the limited- and full-community ambulator groups, which were statistically similar to each other (*p* > 0.05), reported more social participation than household ambulators (both *p* < 0.05).

## Discussion

Our study described the level of balance impairments, the ability to perform ADLs and social participation and examined their relationships with walking speed, among people at the chronic stage of stroke in Burundi, a low-income country. Firstly, we found that most patients experienced poor balance, indicative of a risk of falling, low walking speed, moderate ADL limitations and social participation restrictions. Additionally, household ambulators (low walking speed) displayed major ADL limitations and moderate restrictions in their social participation, and there was a moderate and significant relationship between walking speed and balance. Activities of daily living limitation scores correlated strongly with walking speed, so that slower walking speeds were associated with more limitations. Walking speed, however, did not correlate with social participation.

Impaired balance affects the ability of stroke patients to walk, whether in developed or low-income settings. The low walking speed and poor balance in our sample may be linked to limited early stroke management and access to rehabilitation in low-income settings, as has been shown in previous studies (Bernhardt et al. 2020; Urimubenshi et al. 2018).

In his systematic review, Urimubenshi et al. (2018) concluded that stroke care in Africa fell below the recommended standards and invited African policy makers and health care professionals to combine their efforts to improve access to organised stroke care. Recently, there has been a call to action aimed at developing and delivering cost-effective and equitably accessible rehabilitation services in low- and middle-income countries (Bernhardt et al. 2020). Studies examining the frequency of balance impairments following a stroke (Chang, Gajasinghe & Arambepola 2015; Kossi et al. 2021; Vincent-Onabajo, Musa & Joseph 2018) have reported prevalence rates ranging from 16.7% (Vincent-Onabajo et al. 2018) to as high as 83% (Chang et al. 2015; Kossi et al. 2021). Our finding, that a faster walking speed is related to good balance, is consistent with prior studies (Obembe, Olaogun & Adedoyin 2014; Ojagbemi & Owolabi 2013). Balance disorders are characterised by a short support time and an asymmetrical left versus right step length, which leads to a slow walking speed (Khan & Chevidikunnan 2021). Two major goals of rehabilitation are to enable or improve walking ability and to promote independence in ADLs, both of which are highly dependent on standing balance (Kossi et al. 2019).

Overall, participants in our study showed limitations that affected them performing about half of the assessed ADLs, confirming the persistence of substantial ACTIVLIMs in the chronic phase of stroke. These results are similar to those of a 2019 study carried out in a comparable context of low-income countries (Kossi et al. 2019). In that study, Kossi et al. observed a mean activity level of 55% on the ACTIVLIM-Stroke scale in a sample of 64 stroke survivors, 6 months post-stroke. In 2020, Mwaka-Rutare et al. (2020) reported relatively higher ACTIVLIM-Stroke scores in stroke survivors with a longer post-stroke recovery period (average of 38 months). Their sample’s mean activity level was estimated to be 69%, whereas the overall level observed in our study was 51%. Given the similar socioeconomic contexts of the three studies, the differences in ADL ability levels might be attributed to the baseline clinical characteristics of the samples, such as the disability level. Consistent with this possibility, the mean walking speed of Mwaka-Rutare et al.’s sample (0.9 m/s) was faster than that of both our sample (0.68 m/s) and Kossi et al.’s sample (0.73 m/s).

According to the ICF, walking is a critical component in enabling the performance of ADLs and social participation for stroke patients (Geyh et al. 2004). A limited ability to walk following a stroke restricts patients’ independence and mobility at home and in the community (Schmid et al. 2007). In our study, walking speed was very different between individuals who had different ADL levels. A faster walking speed was associated with better ADL performance scores. Compared with household and limited-community ambulators, participants with full-community ambulation status reported higher ADL levels. Similarly, in their study in the Democratic Republic of Congo, in a socio-economic context similar to that of our study, Mwaka-Rutare et al. (2020) found that faster walking speeds were associated with better performance of basic ADLs. Moreover, our findings seem to be consistent with those of a 2007 study by Schmid et al. (2007) in a high-income context, wherein progression from one walking speed-based category to another was associated with ADL gains.

As in previous studies (Mwaka-Rutare et al. 2020; Schmid et al. 2007), walking speed in our did not correlate well with the participants’ social participation levels. This indicates that a faster walking speed is no longer a priority for the patient in the chronic phase of stroke, but a number of other factors, such as impairment, depression and social support, remain determinants of social participation in both the acute and chronic phases (Elloker & Rhoda 2018; Kossi et al. 2019). However, we obtained a lower optimal walking speed cut-off for distinguishing social participation (0.5 m/s) than was applied by Mwaka-Rutare et al. (2020) and Schmid et al. (2007) (>0.8 m/s). These differences in optimal walking speed cut-offs for social participation may be consequent to our sample being younger on average (52.1 ± 11.4 years) than the samples in these two studies (58.4 ± 12.9 years [Mwaka-Rutare et al. 2020] and 71.0 ± 10.64 years [Schmid et al. 2007]). Young patients are likely to develop better active coping strategies that may contribute to limiting the impact of the reduced walking speed on social participation. Secondly, the lack of a significant association between walking speed and social participation scores, in the chronic stage of stroke, suggests that the optimal cut-off walking speed may be sufficient to enable good social participation, which would support the assumption that social support may be the main factor in enabling social participation without a strict dependence on a faster walking speed (Elloker & Rhoda 2018; Mairami et al. 2020). Finally, the optimal walking speed cut-off of 0.5 m/s found here raises questions about the applicability of Perry et al.’s walking speed classification system in socioeconomic contexts that differ from the context where it was developed (the Los Angeles metropolitan area in the USA). In practice, requirements for safe community walking may differ across locations depending on contextual factors, such as environmental adaptations, the availability and affordability of technical assistance apparatuses and social supports. Further studies should investigate walking speed classification cut-offs that are well suited to an African context.

There are several implications from our findings for clinicians involved in rehabilitation after stroke in low-income settings. Our findings suggest that interventions aimed at improving balance and enhancing walking speed may be useful in promoting long-term ADL ability. Although our results did not confirm a clear association between walking speed and social participation, probably because of socioeconomic contexts, there is some evidence indicating that ADL level may be a key determinant of social participation in the subacute and chronic phases of stroke (Kossi et al. 2019).

## Study strength and limitations

Our study is the first to report the functional recovery of people post-stroke in Burundi, a low-income country. Functional recovery in our sample was evaluated with socioculturally adapted tools and in accordance with the ICF, considering impairment, activity limitations and participation restrictions. However, our findings need to be interpreted in light of some potential limitations. Firstly, because we excluded patients with major cognitive impairments, severe disability and/or with an unclear diagnosis, our findings may not be generalised to the entire stroke population in Burundi. Secondly, despite the statistical normality of the distributions, the unequal numbers of participants in the three groups could have influenced the statistical analyses. In addition, the use of patient-reported outcome tools to assess activity limitations and participation restrictions could generate a recall bias, but we believe that the use of culturally valid Rasch-based questionnaires with very good psychometric properties limits this risk of bias. Finally, Perry’s walking speed classification may not be well suited to the context of low-income countries, given the relatively low average age of their populations and their poorer access to rehabilitation services, compared with that in most developed countries.

## Conclusion

Participants in our study presented major balance impairments with a high risk of falling, slow walking speeds, moderate ADL limitations and moderate restrictions in social participation. It was confirmed that walking speed was associated with balance ability and ADL levels. A faster walking speed was associated with better functional performance, while a moderate speed was sufficient to enable social participation. During the rehabilitation process, particularly in the chronic stage, a special emphasis should be placed on balance and walking speed. Training that targets walking speed and balance can be achieved at a low cost, making it well suited to low-income settings.

## References

[CIT0001] Adoukonou, T., Kossi, O., Fotso Mefo, P., Agbétou, M., Magne, J., Gbaguidi, G. et al., 2021, ‘Stroke case fatality in sub-Saharan Africa: Systematic review and meta-analysis’, *International Journal of Stroke: Official Journal of the International Stroke Society* 16(8), 902–916. 10.1177/1747493021990945 33527885

[CIT0002] Adoukonou, T., Kossi, O., Yamadjako, D. & Agbétou, M., 2018, ‘Restrictions de participation à la vie sociale après un accident vasculaire cérébral chez les sujets âgés au Bénin’, *NPG Neurologie – Psychiatrie – Gériatrie* 18(105), 140–148. 10.1016/j.npg.2018.03.005

[CIT0003] Batcho, C.S., Tennant, A. & Thonnard, J.-L., 2012, ‘ACTIVLIM-stroke: A crosscultural Rasch-built scale of activity limitations in patients with stroke’, *Stroke* 43(3), 815–823. 10.1161/STROKEAHA.111.638965 22223234

[CIT0004] Berg, K.O., Wood-Dauphinee, S.L., Williams, J.I. & Maki, B., 1992, ‘Measuring balance in the elderly: Validation of an instrument’, *Canadian Journal of Public Health = Revue Canadienne De Sante Publique* 83(Suppl 2), S7–S11. 1468055

[CIT0005] Bernhardt, J., Urimubenshi, G., Gandhi, D.B.C. & Eng, J.J., 2020, ‘Stroke rehabilitation in low-income and middle-income countries: A call to action’, *Lancet* 396(10260), 1452–1462. 10.1016/S0140-6736(20)31313-1 33129396

[CIT0006] Bohannon, R.W., 1997, ‘Comfortable and maximum walking speed of adults aged 20–79 years: Reference values and determinants’, *Age and Ageing* 26(1), 15–19. 10.1093/ageing/26.1.15 9143432

[CIT0007] Chang, T., Gajasinghe, S. & Arambepola, C., 2015, ‘Prevalence of stroke and its risk factors in urban Sri Lanka: Population-based study’, *Stroke* 46(10), 2965–2968. 10.1161/STROKEAHA.115.010203 26330444

[CIT0008] Ch’ng, A.M., French, D. & McLean, N., 2008, ‘Coping with the challenges of recovery from stroke: Long term perspectives of stroke support group members’, *Journal of Health Psychology* 13(8), 1136–1146. 10.1177/1359105308095967 18987086

[CIT0009] Durcan, S., Flavin, E. & Horgan, F., 2016, ‘Factors associated with community ambulation in chronic stroke’, *Disability and Rehabilitation* 38(3), 245–249. 10.3109/09638288.2015.1035460 25856203

[CIT0010] Elloker, T. & Rhoda, A.J., 2018, ‘The relationship between social support and participation in stroke: A systematic review’, *African Journal of Disability* 7, 357. 10.4102/ajod.v7i0.357 30349808 PMC6191741

[CIT0011] Ezejimofor, M.C., Chen, Y.-F., Kandala, N.-B., Ezejimofor, B.C., Ezeabasili, A.C., Stranges, S. et al., 2016, ‘Stroke survivors in low- and middle-income countries: A meta-analysis of prevalence and secular trends’, *Journal of the Neurological Sciences* 364, 68–76. 10.1016/j.jns.2016.03.016 27084220

[CIT0012] Faria-Fortini, I., Basílio, M.L., Scianni, A.A., Faria, C.D.C.M. & Teixeira-Salmela, L.F., 2018, ‘Performance and capacity-based measures of locomotion, compared to impairment-based measures, best predicted participation in individuals with hemiparesis due to stroke’, *Disability and Rehabilitation* 40(15), 1791–1798. 10.1080/09638288.2017.1312570 28395524

[CIT0013] Faria-Fortini, I., Polese, J.C., Faria, C.D.C.M. & Teixeira-Salmela, L.F., 2019, ‘Associations between walking speed and participation, according to walking status in individuals with chronic stroke’, *NeuroRehabilitation* 45(3), 341–348. 10.3233/NRE-192805 31796694

[CIT0014] Geyh, S., Cieza, A., Schouten, J., Dickson, H., Frommelt, P., Omar, Z. et al., 2004, ‘ICF core sets for stroke’, *Journal of Rehabilitation Medicine* 44(Suppl), 135–141. 10.1080/16501960410016776 15370761

[CIT0015] Hall, K.S., Gao, S., Emsley, C.L., Ogunniyi, A.O., Morgan, O. & Hendrie, H.C., 2000, ‘Community screening interview for dementia (CSI ‘D’): Performance in five disparate study sites’, *International Journal of Geriatric Psychiatry* 15(6), 521–531. 10.1002/1099-1166(200006)15:6<521::AID-GPS182>3.0.CO;2-F 10861918

[CIT0016] Katan, M. & Luft, A., 2018, ‘Global burden of stroke’, *Seminars in Neurology* 38(2), 208–211. 10.1055/s-0038-1649503 29791947

[CIT0017] Khan, F. & Chevidikunnan, M.F., 2021, ‘Prevalence of balance impairment and factors associated with balance among patients with stroke: A cross sectional retrospective case control study’, *Healthcare* 9(3), 320. 10.3390/healthcare9030320 33805643 PMC7998930

[CIT0018] Kim, A.S., Cahill, E. & Cheng, N.T., 2015, ‘Global Stroke Belt: Geographic Variation in Stroke Burden Worldwide’, *Stroke* 46(12), 3564–3570. 10.1161/strokeaha.115.008226 26486867

[CIT0019] Kossi, O., Agbetou, M., Noukpo, S.I., Triccas, L.T., Dossou-Yovo, D.-E., Amanzonwe, E.R. et al., 2021, ‘Factors associated with balance impairments amongst stroke survivors in northern Benin: A cross-sectional study’, *The South African Journal of Physiotherapy* 77(1), 1559. 10.4102/sajp.v77i1.1559 34693069 PMC8517725

[CIT0020] Kossi, O., Nindorera, F., Adoukonou, T., Penta, M. & Thonnard, J.-L., 2019, ‘Determinants of social participation at 1, 3, and 6 months poststroke in Benin’, *Archives of Physical Medicine and Rehabilitation* 100(11), 2071–2078. 10.1016/j.apmr.2019.03.020 31029652

[CIT0021] Kossi, O., Nindorera, F., Batcho, C.S., Adoukonou, T., Penta, M. & Thonnard, J.-L., 2018, ‘Measuring participation after stroke in Africa: Development of the participation measurement scale’, *Archives of Physical Medicine and Rehabilitation* 99(4), 652–659. 10.1016/j.apmr.2017.10.004 29107042

[CIT0022] Kossi, O. & Thonnard, J.-L., 2018, ‘Tracking changes in participation with participation measurement scale in community-dwelling stroke survivors in Africa’, *Archives of Physical Medicine and Rehabilitation* 99(11), 2238–2243. 10.1016/j.apmr.2018.03.021 29709525

[CIT0023] Lekander, I., Willers, C., Von Euler, M., Lilja, M., Sunnerhagen, K.S., Pessah-Rasmussen, H. et al., 2017, ‘Relationship between functional disability and costs one and two years post stroke’, *PLoS One* 12(4), e0174861. 10.1371/journal.pone.0174861 28384164 PMC5383241

[CIT0024] Mairami, F.F., Warren, N., Allotey, P.A., Mak, J.S. & Reidpath, D.D., 2020, ‘Documenting the impact of stroke in a middle-income country: A Malaysian case study’, *Disability and Rehabilitation* 42(1), 102–113. 10.1080/09638288.2018.1493544 30183424

[CIT0025] Martin, D., 2004, ‘Statistical methods for health care research’, *Physiotherapy Research International* 9(1), 55–56. 10.1002/pri.300

[CIT0026] Mbada, C., Olawuyi, A., Oyewole, O.O., Odole, A.C., Ogundele, A.O. & Fatoye, F., 2019, ‘Characteristics and determinants of community physiotherapy utilization and supply’, *BMC Health Services Research* 19(1), 168. 10.1186/s12913-019-3994-4 30871529 PMC6419371

[CIT0027] Mendis, S., 2013, ‘Stroke disability and rehabilitation of stroke: World Health Organization perspective’, *International Journal of Stroke: Official Journal of the International Stroke Society* 8(1), 3–4. 10.1111/j.1747-4949.2012.00969.x 23280261

[CIT0028] Mwaka-Rutare, C., Perreault, K., Abedi-Mukutenga, P., Masuga-Musafiri, W. & Batcho, C.S., 2020, ‘Activity and participation in stroke survivors in a low-income setting: A cross-sectional study’, *Physiotherapy Research International: The Journal for Researchers and Clinicians in Physical Therapy* 25(4), e1846. 10.1002/pri.1846 32311210

[CIT0029] Obembe, A.O., Olaogun, M.O. & Adedoyin, R., 2014, ‘Gait and balance performance of stroke survivors in South-Western Nigeria – A cross-sectional study’, *The Pan African Medical Journal* 17(Suppl 1), 6. 10.11694/pamj.supp.2014.17.1.3001 PMC3946291 24624242

[CIT0030] Ojagbemi, A. & Owolabi, M., 2013, ‘Predictors of functional dependency after stroke in Nigeria’, *Journal of Stroke and Cerebrovascular Diseases: The Official Journal of National Stroke Association* 22(8), e381–e387. 10.1016/j.jstrokecerebrovasdis.2013.04.015 23680683

[CIT0031] Perry, J., Garrett, M., Gronley, J.K. & Mulroy, S.J., 1995, ‘Classification of walking handicap in the stroke population’, *Stroke* 26(6), 982–989. 10.1161/01.str.26.6.982 7762050

[CIT0032] Pinto, E.B., Nascimento, C., Marinho, C., Oliveira, I., Monteiro, M., Castro, M. et al., 2014, ‘Risk factors associated with falls in adult patients after stroke living in the community: Baseline data from a stroke cohort in Brazil’, *Topics in Stroke Rehabilitation* 21(3), 220–227. 10.1310/tsr2103-220 24985389

[CIT0033] Schmid, A., Duncan, P.W., Studenski, S., Lai, S.M., Richards, L., Perera, S. et al., 2007, ‘Improvements in speed-based gait classifications are meaningful’, *Stroke* 38(7), 2096–2100. 10.1161/STROKEAHA.106.475921 17510461

[CIT0034] Tilson, J.K., Wu, S.S., Cen, S.Y., Feng, Q., Rose, D.R., Behrman, A.L. et al., 2012, ‘Characterizing and identifying risk for falls in the LEAPS study: A randomized clinical trial of interventions to improve walking poststroke’, *Stroke* 43(2), 446–452. 10.1161/STROKEAHA.111.636258 22246687 PMC3265675

[CIT0035] Urimubenshi, G., Cadilhac, D.A., Kagwiza, J.N., Wu, O. & Langhorne, P., 2018, ‘Stroke care in Africa: A systematic review of the literature’, *International Journal of Stroke: Official Journal of the International Stroke Society* 13(8), 797–805. 10.1177/1747493018772747 29664359

[CIT0036] Van der Zee, C.H., Visser-Meily, J.M.A., Lindeman, E., Jaap Kappelle, L. & Post, M.W.M., 2013, ‘Participation in the chronic phase of stroke’, *Topics in Stroke Rehabilitation* 20(1), 52–61. 10.1310/tsr2001-52 23340071

[CIT0037] Vincent-Onabajo, G., Musa, H.Y. & Joseph, E., 2018, ‘Prevalence of balance impairment among stroke survivors undergoing neurorehabilitation in Nigeria’, *Journal of Stroke and Cerebrovascular Diseases: The Official Journal of National Stroke Association* 27(12), 3487–3492. 10.1016/j.jstrokecerebrovasdis.2018.08.024 30205998

[CIT0038] Wang, Y., Mukaino, M., Ohtsuka, K., Otaka, Y., Tanikawa, H., Matsuda, F. et al., 2020, ‘Gait characteristics of post-stroke hemiparetic patients with different walking speeds’, *International Journal of Rehabilitation Research* 43(1), 69–75. 10.1097/MRR.0000000000000391 31855899 PMC7028468

